# High‐Risk HPV in Men: A Hidden Threat to Public Health?

**DOI:** 10.1002/rmv.70115

**Published:** 2026-02-14

**Authors:** Keesha L. Coker, Ethan L. Morgan

**Affiliations:** ^1^ School of Life Sciences University of Sussex Brighton UK; ^2^ Tumour Virology Group The Cyprus Institute of Neurology and Genetics Nicosia Cyprus

**Keywords:** HPV, public health, vaccination

## Abstract

High‐risk human papillomavirus (HR‐HPV) infection is a leading cause of several cancers, including those of the genital and oropharyngeal regions. While public health efforts have largely focused on women due to its link to cervical cancer, HPV also poses significant risks to men, particularly in the oropharyngeal regions. HR‐HPV prevalence in men is high, with global estimates of 21% for male genital infections. While the HPV vaccination programme has expanded to include boys, challenges remain, including a decline in vaccine uptake due to COVID‐19 disruptions, vaccine hesitancy, and misinformation. These barriers hinder the full potential of vaccination efforts. Furthermore, HPV transmission is complex and multifactorial, making it difficult to track, while its prevalence, clearance, and persistence vary based on factors such as sexual behaviour and immune status. Additionally, data from lower socio‐economic regions is limited, highlighting a critical gap in research. Specific data on these epidemiological characteristics for male patients is lacking, prompting the need for gender‐balanced approaches. Here, we explore the prevalence, risks, and public health implications of high‐risk HPV (HR‐HPV) in men. We suggest a more inclusive approach to HPV prevention, emphasising the need for targeted vaccination and screening programs for men. A gender‐neutral approach is crucial to reducing the global burden of HPV‐related diseases and moving closer to the goal of eradicating HPV infections worldwide.

**Abbreviations**
ASIR: Age‐Standardised Incidence Rate
ASMR: Age‐Specific Mortality Rate
HIV: Human immunodeficiency virus
HNSCC: Head and neck squamous cell carcinoma
HPV: Human papillomavirus
HR: High risk
MSM: Men who have sex with men
STI: Sexually transmitted infection

## 1 Introduction

Human papillomavirus (HPV) is one of the most prevalent sexually transmitted infections worldwide, with over 80% of sexually active individuals projected to contract the virus at least once in their life [1]. Of the 400 identified HPV types, high‐risk HPV (HR‐HPV) types, such as HPV16 and HPV18, are responsible for nearly 5% of all cancers globally, including cervical cancer, oropharyngeal cancer and several anogenital cancers (vaginal, vulvar, penile and anal cancer) [2]. While the association between HPV and cervical cancer in women is well established, its impact on men's health has garnered increasing attention in recent years. In particular, the rising incidence of oropharyngeal cancers ‐ now the most common HPV‐related cancers in some regions [3] ‐ underscores the critical need to address HPV as a shared public health concern. Despite this, the impact of HR‐HPV on male populations remains poorly understood, revealing significant gaps in research and prevention efforts.

Public health initiatives have historically focused on HPV in women due to the high burden of cervical cancer and the availability of screening and vaccination programs [4]. These efforts have markedly reduced cervical cancer rates, particularly in high‐income countries. However, this female‐centric approach has overlooked the risks HPV poses to men. Current research has primarily examined high‐risk male populations, such as men who have sex with men (MSM) and immunocompromised individuals, leaving the general male population inadequately represented. As a result, men not only face heightened risks of HPV‐related cancers but also contribute to the continued transmission of the virus, exacerbating its public health impact.

The lack of gender‐neutral strategies, including inclusive vaccination campaigns and screening protocols for men, compounds this issue. Although the HPV vaccine is highly effective in preventing infection and related cancers, its uptake remains inconsistent across male populations and underserved regions, often hindered by limited awareness and access [5]. The COVID‐19 pandemic has further disrupted vaccination programs, reducing coverage for both sexes and fuelling vaccine hesitancy [6]. Additionally, the absence of standardised HPV screening for men prevents early detection of infections or precancerous conditions, leaving many unaware of their risks [7]. This inequity in public health policy not only neglects men's health but also undermines the broader goal of eradicating HPV and its associated diseases by perpetuating the circulation of infection.

Here, we seek to address critical gaps in the understanding of HR‐HPV in men by highlighting its prevalence, epidemiology, and associated health risks. By challenging the perception of HPV as a women's health issue, we advocate for gender‐neutral vaccination and screening protocols, aiming to enhance global prevention strategies and public health outcomes. Ultimately, we aim to provide actionable recommendations for equitable and inclusive HPV prevention efforts worldwide.

## 2 Methods

A systematic literature search was performed to identify studies examining the prevalence of HPV in men, its association with high‐risk HPV types and related cancers in men, as well as evaluate vaccinology and current HPV related public health programs in men. The search was conducted using the PubMed database, focussing on publications from 2010 onwards. The search strategy was based on predefined keywords and MeSH terms relevant to the research question. Key search terms included: ‘High‐risk HPV in men’, ‘HPV‐associated cancers in men’, ‘oropharyngeal cancer’, ‘HPV epidemiology in men’, ‘HPV vaccination in men’ and ‘HPV in men versus women’. Boolean operators (AND, OR and NOT) were used to refine and combine search terms yielding as many relevant studies as possible.

The search was restricted to articles published in English, focussing on human studies involving male populations. Studies were included if they assessed high‐risk HPV types in men and investigated HPV‐related cancers. Comparisons to male figures were made where relevant using studies focused on women. Studies were excluded if they involved animal or in vitro research or primarily examined low‐risk HPV types.

## 3 HPV Infection and Transmission

The primary drivers of HR‐HPV induced oncogenesis are the viral oncogenes E6 and E7, which manipulate host cell signalling pathways to promote proliferation and survival [8, 9]. Furthermore, HR‐HPV genomes can also integrate into the host genome, resulting in uncontrolled expression of E6 and E7 and driving oncogenesis [10, 11]. While most HPV infections are transient and resolve spontaneously within 1–2 years after exposure [12], persistent infection with HR‐HPV is a critical factor in the development of pre‐cancerous lesions that can progress to cancer [13].

**FIGURE 1:**
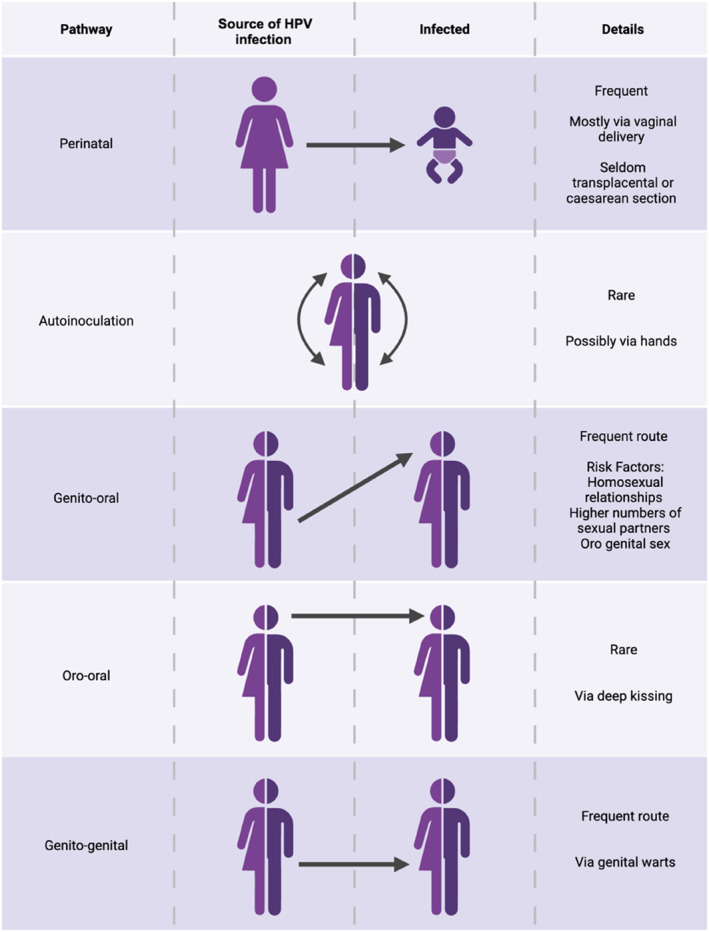
Primary transmission routes of HPV. Illustration of the primary transmission routes of human papillomavirus (HPV), depicting the sources of infection and providing relevant details on the frequency of transmission. Adapted from [31].

HPV is primarily transmitted through direct skin‐to‐skin contact during sexual activities although its ease and diversity of transmission is evidenced by its spread through non‐penetrative sexual contact, such as genital‐to‐genital contact as depicted in Figure 1 [14, 15]. An important but often overlooked route of transmission is oro‐genital contact, where the virus is transferred from the genital area to the oropharynx. This pathway has gained attention due to the rising rates of oropharyngeal cancers linked to HPV [16, 17] particularly among men, where in some regions, oropharyngeal cancers now surpass cervical cancer in annual incidence [18].

HPVs adaptability is further seen through autoinoculation between anatomical sites, though this remains debated [19]. Additionally, studies show inter‐site transmission between partners, with identical HPV types detected across multiple sites, demonstrating seamless spread between site and partners [20].

Other research has identified factors influencing HPV transmission and persistence, including number of sexual partners [21], HIV status [22], circumcision status [23, 24], partner HPV status, and condom use [25]. Transmission is expectedly higher with HPV‐positive partners [26]. Such variability highlights the complex interplay of host and environmental factors shaping HPV epidemiology. HPV's high transmissibility presents major challenges for kerbing its spread. Contemporary sexual behaviours, such as casual partnerships driven by dating apps [27, 28] and the growing prevalence of oral sex may be the cause of amplified transmission and contribution to rising HPV‐related oropharyngeal cancers, particularly in men [29, 30]. Inconsistent condom use paired with limited awareness of risks beyond cervical cancer further facilitate spread across populations, underscoring the need to address this as a universal public health concern requiring strategies that account for its unpredictable nature.

## 4 HPV Clearance

HPV infection exhibits a dynamic natural history, with most infections clearing spontaneously but some persisting long‐term, depending on a combination of viral, host, and environmental factors. HPV clearance rates are influenced by factors such as age, immune competence, and co‐factors like smoking or coinfections [32, 33], as well as demographic, biological, and anatomical factors [34]. While persistence increases disease risk, timely clearance mitigates long‐term outcomes, including HPV‐related cancer development. The HPV Infection in Men (HIM) Study, a multinational cohort of 4085 HIV‐negative men aged 17–80, examined HPV incidence and clearance. The study found variations across genotypes, with a median clearance time of 7.52 months (95% confidence interval (CI) 6.80–8.61) for any HPV and 12.19 months (95% CI 7.16–18.17) for HPV16, reflecting its immune evasion and oncogenic potential [35, 36]. These findings emphasise the need to monitor high‐risk types, particularly in male populations, due to their role in cancers of the penis, anus, and oropharynx.

HPV clearance rates differ between sex, HPV type, site of infection and study. Genital HPV infection in men had a median clearance time of 5.9 months, with 75% resolving within 12 months [35]. In women, where high‐risk types like HPV16 can persist longer, there was a median clearance time of 11 months [37]. At non‐genital sites, such as the oropharynx, men show higher persistent HPV infections, where prevalence is nearly three times higher in men (10.1%) than in women (3.6%), posing significant challenges due to their association with oropharyngeal cancers [38]. Age impacts HPV clearance differently in men and women. Research suggests older men may clear genital HPV infections faster, potentially due to reduced exposure to new infections and immune adaptations [39]. Conversely, older women, especially post‐menopause, experience reactivation of HPV infections and seemingly slower clearance [40].

Geographical and socioeconomic disparities significantly influence HPV clearance. High‐income regions often report shorter clearance durations due to earlier detection, better healthcare follow‐up, and broader vaccination coverage. In contrast, individuals in low‐ and middle‐income countries may experience delayed diagnosis and limited access to screening or treatment, contributing to persistence and higher progression risk. These can also manifest as intra‐regional sex disparities, where women may access routine reproductive health services that improve early detection, while men often face stigma or limited awareness of HPV risk, leading to not only undiagnosed or persistent infections but imbalanced healthcare outcomes. Once again factors such as circumcision prevalence, hygiene practices, smoking, and coinfections (e.g., HIV or other STIs) further shape clearance patterns across populations. However, this variability in clearance and persistence remains an under‐researched area of HPV natural history. The HIM Study's robust design and multinational scope have helped establish reference points for HPV clearance timelines and clarified sex‐specific patterns. However, a more globally inclusive approach is needed to account for underrepresented populations and variable regional contexts. Whilst the implementation of screening programs in men will aid in this endevour, detailed tracking of HPV persistence in infected individuals at the molecular level are also required.

## 5 Prevalence of HR‐HPV in Males

The epidemiology of HR‐HPV in males has been significantly under‐researched compared to women, with most prior research limited to high‐risk groups such as men who have MSM and individuals with HIV. This narrow focus has hindered the development of comprehensive prevention strategies targeting the general male population.

**FIGURE 2:**
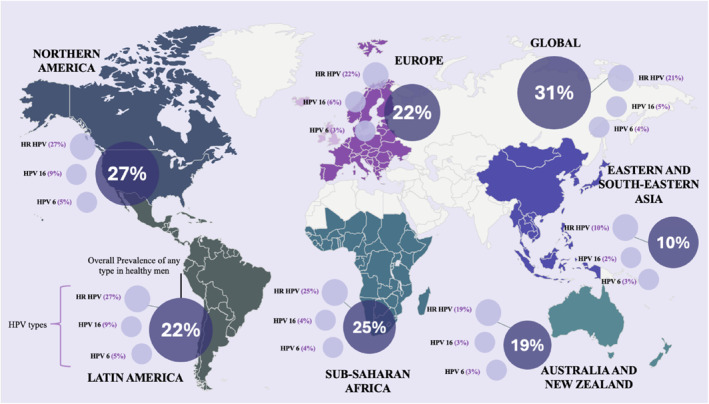
Global Percentage Distribution of Genital HPV Prevalence in Men. The map highlights the regional prevalence of any HPV type, with additional data illustrating the percentage distribution of HR‐HPV types and specific HPV types 16 and 6. Created based on data from [41].

To address this gap, Bruni et al. (2023) [41] conducted a systematic review estimating male genital HPV prevalence, including over 44,000 men from 65 studies across 35 countries, one of the most comprehensive analyses to date. Nonetheless, it remains modest in scale compared with Bruni et al.’s 2010 study [42] in women, which evaluated HPV prevalence in more than one million participants. Bruni et al.’s 2023 study samples a diverse range of cohorts including university students, military personnel, and asymptomatic men while excluding high‐risk groups to establish a critical baseline for assessing the impact of gender‐neutral vaccination programs in the future. Findings showed a global HPV prevalence of 31% in men, with HR‐HPV at 21%. HPV16 emerged as the most common type in four of the five global regions analysed, as depicted in Figure 2, with a global prevalence of 5%, solidifying its major public health impact in both sexes [43].

HPV infection is widespread across the male lifespan, with prevalence peaking among individuals aged 25–29, particularly for HPV16. Elevated rates were also observed in males aged 15–19, a finding consistent with transmission shortly after sexual debut, a pattern similarly seen in females [44]. Regionally, aggregated data reinforced this global trend, with a consistent peak in HPV prevalence across all categories between the ages of 20 and 29. Sub‐Saharan Africa and Northern America reported the highest prevalence rates, particularly among men aged 25–29, while Eastern and South‐Eastern Asia exhibited substantially lower rates. These regional differences may reflect variations in sexual behaviours, cultural practices, healthcare access, and HPV vaccination uptake much like those to do with clearance.

In most regions HPV prevalence showed a gradual decline after the late twenties, however Europe and Latin America and the Caribbean exhibited a secondary rise among men aged ≥ 50, a bimodal pattern also seen in female populations [45], and may reflect biological and behavioural shifts with age, including immunosenescence and changes in sexual activity. In North America, prevalence remained elevated extended into ages 30–39, particularly for any HPV type and HR‐HPV. Methodological differences also influenced estimates, with studies sampling multiple genital sites (e.g., glans, coronal sulcus, penile shaft) reported consistently higher prevalence than single‐site studies [46], and broader HPV genotype panels naturally yield higher rates. These inconsistencies in methodology underscore the need for standardised sampling and testing protocols to improve comparability and global burden estimates.

Geographical and socioeconomic factors also shaped prevalence patterns, mirroring trends in clearance. According to the 2023 Bruni et al. genital prevalence study [41], high‐income countries reported higher pooled prevalence for any HPV, HR‐HPV, and HPV16 (e.g., 34% vs. 28% for any HPV). These differences likely reflect greater access to diagnostics capacity and surveillance. Conversely, low‐ and middle‐income countries face higher infection risk due to limited vaccination, healthcare access, and sexual health education. Nearly half of the dataset originated from Europe and the Americas, underscoring underrepresentation of low and middle‐income regions. This imbalance constrains global HPV burden estimates and risks perpetuating disparities in prevention and treatment. Addressing these gaps through targeted investment in research, education, and healthcare infrastructure is essential to reducing HPV‐related inequities.

Progress has been made in closing the long‐standing gap in oral HPV data among healthy men. Alemany et al. (2025) [47] conducted a multinational study of 7674 adults (16–80 years) using data from dental clinics in five high‐income countries, with males comprising nearly half the cohort. Oral HPV, particularly HR‐HPV types, were consistently more prevalent in men. In the UK, for instance, 15.0% of men tested positive for any oral HPV compared to 6.8% of women, and HR‐HPV was found in 4.5% of men versus 1.2% of women. Risk factors included older age, marijuana use, and a high number of lifetime oral sex partners, while conditions such as periodontitis may also have contributed to the variation of prevalence across countries. As one of the largest recent studies on male HPV, this investigation highlights older men as having the highest oral HPV burden, parallelling rising rates of HPV‐related oropharyngeal cancer [17]. The findings represent missed opportunities for prevention as well as the need for targeted vaccination and screening beyond adolescence. Although focused on high‐income countries, the study supports expanding oral HPV research globally.

## 6 High‐Risk HPV‐Related Disease

Cervical cancer remains the most widely recognised HPV‐related malignancy in women, with over 90% of cases attributed to HR‐HPV types, primarily HPV16 and HPV18 [48, 49]. Screening and vaccination programs have reduced incidence in many high‐income countries [50, 51], yet cervical cancer continues to impose a substantial global health burden. It remains one of the leading causes of cancer‐related mortality among women, especially in low‐ and middle‐income countries where access to preventive measures is limited [52]. In 2020, approximately 604,127 new cases of cervical cancer and 341,831 deaths were reported [4], emphasising the need for further research into causes and prevention methods.

While cervical cancer has long dominated HPV research, HPV‐associated cancers in men, particularly head and neck squamous cell carcinoma (HNSCC), specifically oropharyngeal cancer, have emerged as a critical public health concern. HNSCC encompasses malignancies of the oral cavity, pharynx, and larynx. Oropharyngeal cancers affect the tonsils, base of the tongue, and soft palate and are the most rapidly rising HPV‐associated malignancy in men [53, 54]. In the USA, 70%–80% of these cancers are HPV‐positive, predominantly HPV16, surpassing traditional risk factors like tobacco and alcohol [17, 55]. Between 1988 and 2004, HPV positive oropharyngeal cancers increased by 225%, with men disproportionately affected [3, 56].

Anal cancer is another HPV‐related malignancy affecting both sexes, with higher overall incidence in women although high‐risk men, particularly MSM and individuals with HIV, face markedly elevated rates [57]. Among MSM with HIV, incidence exceeds 131 per 100,000 annually, a rate comparable to pre‐screening cervical cancer in women [58, 59, 60]. These findings cement the need for research in both high‐risk men and the general population. Evidence also suggests that genital HPV infections may act as precursors to anal cancer through inter‐site transmission [61], reinforcing the need for comprehensive HPV prevention and detection strategies.

HPV is also implicated in around 50% of invasive penile cancers [62], though estimates vary by study design and cancer subtype [63, 64]. Globally, penile cancer remains rare, with a 2020 Age‐Standardised Incidence Rate (ASIR) of 0.80 and a Age‐Specific Mortality Rate (ASMR) of 0.29 per 100,000, equating to 36,068 new cases and 13,211 deaths [65]. Incidence has risen in some regions, including a 21% increase in England between 1979 and 2009 [66], and GLOBOCAN projects a > 56% rise by 2040 [67]. The burden is disproportionately higher in low‐ and middle‐income countries, which account for nearly half of global deaths associated with the disease and show ASIR and ASMR rates nearly double those of high‐income regions [65].

Beyond cancer, HPV has also been implicated in reproductive health. Persistent genital HPV has been linked to reduced sperm quality in men [68] and to endometrial inflammation and impaired implantation in women [69]. HPV is also associated with vulvar and vaginal cancers, which, though rare, are increasing in certain populations, likely due to rising HPV prevalence and delayed vaccination uptake [65, 70].

## 7 Global Vaccination Strategies

The introduction of three HPV vaccines ‐ Gardasil (2006), Cervarix (2007), and Gardasil 9 (2014) ‐ has represented a major leap forward in combating HPV‐related disease [71]. Gardasil, a quadrivalent vaccine, protects against HPV types 6, 11, 16, and 18, while Cervarix, a bivalent vaccine, protects against HR‐HPV types 16 and 18. Gardasil 9 expanded coverage to nine HPV types, protecting against HPV types that cause nearly 90% of HPV‐associated disease [72]. Studies show substantial declines in HPV prevalence, genital warts, and cervical precancerous lesions following vaccination [73]. In MSM, quadrivalent vaccination reduced anal HPV by 76% and penile infection by 52%, with notable decreases in HPV16/18 [74]. Vaccination in men also lowers oral HPV16 prevalence, a marker for oropharyngeal cancer [75].

Since the introduction of the first HPV vaccine in 2006, global programs have focused on females due to heavy burden of cervical cancer, which is caused by HPV in over 90% of cases [48]. This female‐centric approach, based on expected herd immunity [76, 77], delayed male vaccination, with recommendations only being made in some regions from 2009 [78]. Implementation has remained uneven, exemplified by the 10‐year delay before boys were vaccinated in the UK [79]. Nevertheless, several organisations had previously advocated for a gender‐neutral vaccination approach, including the UK HPV Action Group, which published policy‐focused evidence supporting universal vaccination prior to its adoption by national health authorities in 2019 [80].

Men's direct risk from HPV‐related cancers was largely overlooked in the early stages of vaccine implementation. In the UK, vaccination is offered to children aged 12 to 13 to establish immunity before potential exposure to the virus, often linked to sexual debut. This approach aligns with similar programs in other countries. This strategy has driven substantial declines in cervical cancer, with recent data showing effectiveness in women exceeding projections [4, 81]. For instance, cervical cancer mortality rate in US women under 25 has fallen to < 0.01 per 100,000 person‐years, alongside a long‐term drop from > 60 per 100,000 in the 1950s to < 15 by 2019–2021. Yet, the absence of early gender‐neutral vaccination has left a generation of men unvaccinated and at elevated risk of HPV‐related disease.

These challenges have been exacerbated by the COVID‐19 pandemic, which disrupted healthcare services globally, including vaccination programs [6]. A marked decline in HPV vaccine uptake has been observed since the pandemic's onset, mirroring trends for other vaccines [82]. This decline stems from disruptions to routine healthcare, reduced school access (key to HPV vaccination programs), and the redirection of public health resources to COVID‐19 management. The pandemic has also fuelled vaccine hesitancy, partly due to distrust surrounding COVID‐19 vaccines, which has spilled over to HPV and other vaccinations. These disruptions risk reversing progress in HPV prevention and widening vaccination gaps, particularly for men. A global, comprehensive, gender‐neutral vaccination strategy is essential to address these gaps, including proactive vaccination of boys from adolescence and educational campaigns to raise awareness of HPV risks for both sexes. Such measures would not only protect men from HPV‐related cancers but also reduce HPV's overall public health burden, creating a more equitable and effective prevention strategy worldwide.

## 8 Screening and Prevention Practices

Currently, there are no routine HPV screening protocols for men. Testing usually occurs when symptoms such as genital warts are present, when a partner has an abnormal cervical screen, or incidentally in sexual health clinics. The absence of standardised screening for asymptomatic men leaves major gaps in prevention and early detection [7].

Various methods for HPV detection in men have been explored to date. Penile swabs, which involve sampling from the glans, shaft, and coronal sulcus, have demonstrated higher HPV detection rates. Anal swabs and anal cytology are also utilised, primarily among high‐risk populations such as MSM or HIV‐positive men [83]. Additionally, urethral brushing, semen sampling, and self‐sampling methods have shown promise. Self‐collected genital samples in women have been found to achieve accuracy comparable to clinician‐collected samples, suggesting potential for broader applications [84]. However, these approaches remain unstandardised, and further research is needed to refine their sensitivity, reliability, and acceptability for widespread implementation.

In contrast, women benefit from routine, proactive HPV screening measures, including Pap smears and HPV DNA testing. These methodologies offer different sensitivities; Pap smears have a sensitivity of 55.4% (95% CI, 33.6–77.2) and a specificity of 96.8% (95% CI, 96.3–97.3) for the detection of high‐grade cervical lesions, whereas HPV DNA testing demonstrates higher sensitivity (94.6% (95% CI, 84.2–100.0)) and lower specificity (94.1% (95% CI, 93.4–94.8) [85]. These screening methods have been shown to reduce cervical pre‐cancer and invasive cervical cancer incidence by upto 70% by detecting pre‐malignant lesions or high‐risk HPV infection before progression to invasive disease, enabling early clinical intervention [86]. Vaccination programs targeting HR‐HPV strains further bolster prevention strategies for women [51, 87].

These limitations in HPV screening and prevention are more pronounced in low‐ and middle‐income countries (LMICs), where reported HPV prevalence is strongly influenced by healthcare infrastructure. In LMICs, HPV screening coverage is low, reflecting constraints in healthcare funding and laboratory availability, resulting in late diagnosis of cervical cancers [88]. HPV surveillance systems in LMICs are also often fragmented, with HPV data derived from small, urban‐based studies, cancer registries with incomplete population coverage, or research cohorts not integrated into national health reporting structures. This limits longitudinal monitoring of HPV infection and associated disease. In parallel, HPV vaccination coverage remains lower in LMICs [89] due to financial constraints, vaccine availability, and reduced programme reach.

The absence of standardised HPV testing for men is especially concerning given the prevalence of asymptomatic carriers, who may unknowingly transmit the virus to their partners. Rising rates of HPV‐related cancers in men highlight the critical need for improved detection and prevention strategies. A comprehensive public health approach that combines standardised screening protocols for men with expanded vaccination initiatives could dramatically reduce HPV transmission and associated disease burdens, replicating the success achieved in women.

## 9 Implementation

The implementation of screening for HR‐HPV in men presents significant practical challenges, such as the lack of a specific HPV test for men as previously discussed. If routine screening were to be considered, it would be most appropriately targeted towards high‐risk groups, including men who have sex with men, immunocompromised individuals, and men who report sexual contact with HPV‐positive partners.

Available HPV DNA tests are primarily for high‐risk genotypes; however, HPV infection alone does not constitute disease. Therefore, implementation should focus on the identification of pre‐malignant disease or malignancy rather than HPV positivity in isolation. Individuals who screen positive for HPV should have access to further clinical assessment or treatment, as is the case with women who test positive. Educational resources and councilling should also be provided so that HPV positive individuals can understand all potential outcomes.

Given the asymptomatic nature of HPV in men before the onset of cancer, the feasibility of providing widespread screening, counselling and follow‐up within national healthcare systems, such as the NHS, remains uncertain due to resource and workforce constraints. Screening would likely be delivered through specialist health clinics or private clinical test centres, where appropriate expertise and follow‐up services would be available.

## 10 Conclusion and Future Prospects

In conclusion, HPV has the potential to persist in the male population, leading to a variety of cancers in the genital and head/neck regions. The persistence and transmission of HPV are shaped by multiple factors, including sexual behaviours, cultural norms, and individual health conditions such as age and immune status. These findings highlight the complexities of HPV epidemiology and natural history in men and underscore the existing gaps in understanding and addressing its full impact.

There is a significant lack of comprehensive data on HPV in the general male population. Social stigma, limited awareness, and reactive testing protocols contribute to underreporting and hinder a full understanding of the true burden of the virus. As a result, many men remain at risk, with HPV‐related cancers like oropharyngeal cancer emerging as critical public health concerns. These statistics reinforce the urgent need for inclusive prevention strategies that extend to men.

Given these findings, addressing the HR‐HPV burden in men requires immediate and comprehensive action. First, HPV testing for men must shift from reactive approaches to more structured, evidence‐informed screening focused on high‐risk groups. Such targeted approaches would enable earlier detection of HPV‐related malignancies, improve clinical outcomes, and provide data to better understand male HPV epidemiology. Concurrently, vaccination strategies should expand to a gender‐neutral approach, ensuring equitable access for boys and men and closing gaps left by earlier initiatives. Catch‐up vaccination for high‐risk populations is particularly important given evidence of higher infection prevalence, longer persistence, and elevated cancer risk in these groups.

Public health policies must prioritise rebuilding vaccine confidence and providing accurate information to the public, addressing misinformation, stigma, and pandemic‐related disruptions to HPV prevention services. Future research should explore HPV's role in non‐cancerous conditions and male reproductive health, examine inter‐site transmission, and include underrepresented populations such as older and heterosexual men. By focussing on structured high‐risk screening, equitable vaccination, and targeted public engagement, the global burden of HR‐HPV in men can be realistically reduced, ensuring that both men and women benefit from advances in HPV prevention.

## Author Contributions

K.L.C: conceptualisation, literature review, initial draft writing, reviewing and editing. E.L.M.: project supervision, writing, reviewing and editing. Both authors have read and agreed to the published version of the manuscript.

## Funding

The authors have nothing to report.

## Conflicts of Interest

The authors declare no conflicts of interest.

## Data Availability

Data sharing not applicable to this article as no datasets were generated or analysed during the current study.
